# Milk somatic cells, factors influencing their release, future prospects, and practical utility in dairy animals: An overview

**DOI:** 10.14202/vetworld.2018.562-577

**Published:** 2018-05-02

**Authors:** Mohanned Naif Alhussien, Ajay Kumar Dang

**Affiliations:** Lactation and Immuno-Physiology Laboratory; ICAR-National Dairy Research Institute, Karnal, Haryana - 132 001, India

**Keywords:** dairy animals, factors influencing, milk, somatic cells release, utility

## Abstract

Milk somatic cells (SCs) are a mixture of milk-producing cells and immune cells. These cells are secreted in milk during the normal course of milking and are used as an index for estimating mammary health and milk quality of dairy animals worldwide. Milk SC is influenced by cow productivity, health, parity, lactation stage, and breed of an animal. Any change in environmental conditions, poor management practices, and also stressful conditions significantly increases the amount of SC coming in milk. Better hygiene and proper nutrition help in reducing milk SC. Milk with low SC means better milk products with a longer shelf life. The present review describes the role of SCs (both secretory and immune) in milk, their role in maintaining the integrity of the mammary gland, and factors affecting their release in milk. This information may help to reduce milk somatic cell counts (SCCs) and to establish differential SCC standards.

## Introduction

**Figure-1:**
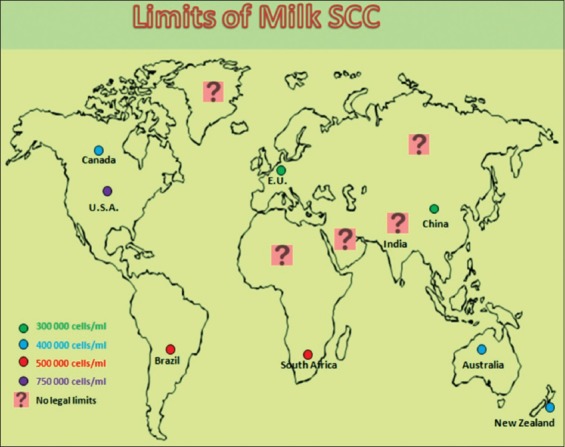
Diagrammatic representation of the worldwide limits of milk somatic cell counts in cows. Source: Alhussien and Dang

The udder or mammary gland has evolved in all the mammalian species to nourish their young one. However, through genetic selection and advances in milking technology, the mammary gland is now producing more milk than a calf can consume and far greater quantities than the original organ were designed to accommodate. The selection of dairy animals for greater milk production and the removal of milk by machine milking impose unnatural stress on the bovine udder. This has increased the chances of mammary infections in these animals. To defend against the mammary infections, somatic cells (SCs) are released into the milk. These cells not only fight infection but also repair tissue damage. All the developed countries are using milk somatic cell counts (SCCs) as a marker to monitor the prevalence of mastitis in dairy herds, as an indicator of raw milk quality to processors, and also as a more general indicator of the hygienic conditions of milk production on farms [1,2]. Out of all the different milk quality screening tests, estimation of milk SCs is the most effective method to detect the subclinical form of mastitis. In the European Union, China, New Zealand, Australia, Switzerland, and Canada, the legal bulk milk SCC (BMSCC) limit is 3-4×10^5^ cells/mL; in South Africa and Brazil, 5×10^5^ cells/mL; and in the USA, 7.5×10^5^ cells/mL (Figure-1). In developed countries, processors pay a premium for low count milk as this milk has more technological traits and longer shelf life. However, in developing countries like India, milk is still sold based on its fat percentage. However, nowadays, geographical boundaries for dairy products are expanding and to compete in the international market; there is a need to set a level or standard of milk SCC in all milk exporting countries. The present review discusses the release of SC in milk, factors affecting their release, estimation of milk SCC in cows, buffaloes, ewes, goats, and camels, and their future use in dairy industry and for other experimental purposes.

## Cow Mammary Gland

Mammals have derived their name from the word “mammary” and the mammary gland is the milk-producing gland present in female mammals. It is a modified sweat gland and consists of the mamma and the teat. It is undeveloped in both the male and female at birth and begins to develop as a secondary sex characteristic in females when they attain puberty. With the birth of the first young one, the mammary gland attains its full size and starts producing milk. The basic components of a lactating mammary gland are the alveoli (hollow cavities) lined with milk-secreting cuboidal cells known as epithelial cells. These are surrounded by myoepithelial or muscle cells which contract under the influence of hormone oxytocin to drain out the milk from the alveolar lumen into the ducts. Alveoli join to form groups known as lobules, and each lobule has a lactiferous duct that drains into openings in the teat. Milk is continuously synthesized in the alveolar area and is stored in the alveoli, milk ducts, and teat cistern between milkings.

Mammary gland has various cellular, anatomical, and humoral defense mechanisms which help in preventing the invasion and establishment of pathogens in the gland [3]. Pathogens usually gain entry to the quarter through the teat canal before, during, and after lactation periods. During dry periods and between milkings, teat canal is sealed by a keratin plug which is formed from the stratified squamous epithelial lining within the duct. This forms an effective physical and microbicidal barrier against invading microorganisms. However, damage to the plug can temporarily or permanently increase the penetrability of the teat canal, thus increasing the chances of mammary infections. Milk SC, particularly the milk leukocytes, increases significantly once there is a bacterial infection.

## What is Milk SCC?

The term SCs mean body-derived cells that are normally present at low levels in milk. The majority of these cells in normal milk are cells from the udder secretory tissue (epithelial cells), and some are leukocytes (white blood cells). SCs represent the second line of defense, the first being the anatomical and chemical barriers of the teat apex and canal in the mammary gland. The epithelial cells in milk result from the desquamation of the mammary epithelium of the alveoli and the ducts. The presence of such cells coming in milk is a normal physiological phenomenon and is necessary for the regeneration of normal epithelia. Majority of exfoliated epithelial cells present in milk are viable and exhibit characteristics of fully differentiated alveolar cells [4].

White blood cells coming in milk are all blood-borne SCs and serve as a part of the defense system. Their primary function is to fight disease and assist in repairing damaged tissue. Any intramammary infection (IMI) (mastitis) leads to an increase in these cells in milk and indicates poor hygiene of the produced milk. The SCC is quantified as the number of cells per ml of milk. When the amount of SCC is around 1 lakh, it indicates that an animal is unaffected [5]. Cows and buffaloes having more than 200,000 SCC/ml of milk are likely to be infected in at least one quarter [6,7]. As the cell count elevates, it is directly related to the severity of infection and the number of infected quarters in a cow.

## Mechanism of Release of SCC in Milk

Milk is continuously being produced in a lactating mammary gland by the milk-secreting epithelial cells. These epithelial cells are lined by blood vessels and take up various milk precursors from blood, thus synthesizing and releasing milk into the alveolar lumen. Whenever there is any breach of the mammary barriers by an invading pathogen, it causes tissue damage and releases a variety of different chemical compounds into the mammary system. The epithelial cells initiate the first step in the body by limiting the extent of infection and activate the mammary immune system. Two components, the cellular (leukocytes) and the humoral (immunoglobulin) component, play a significant role in reducing and eliminating these infections. The cellular component includes the white blood cells (neutrophils, lymphocytes, and macrophages) normally present in a healthy udder and others that are activated by the immune system of the mammary gland [7]. However, humoral components are indicated by macromolecules such as antibodies, complement proteins, and antimicrobial peptides.

**Figure-2:**
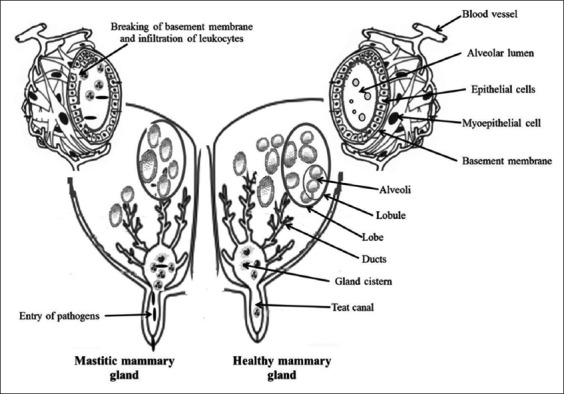
Diagrammatic representation showing both healthy and mastitis mammary gland. The invasion of pathogens to the internal tissues of mammary gland stimulates the trafficking of various immune cells to the site of inflammation which results in elevation of somatic cell counts in the secreted milk. Source: Alhussien and Dang

Leukocytes are always circulating and keep a vigil because with every milking, the teat sphincter is opened, thus making the mammary gland under threat of infections. Whenever the resident macrophages in the mammary gland identify the presence of some harmful bacteria, they signal to other leukocytes and immune cells about such infection [8]. Consequently, more immune cells get recruited to the area of infection, and hence, the SCC in milk increases. Phagocytic cells, namely, neutrophils and macrophages, after sensing the bacteria, tend to lock on and envelope the bacteria and this process is referred to as phagocytosis. Once bacteria are internalized, the phagocytic cells proceed to destroy them by releasing some enzymes capable of digesting bacterial components. During inflammation, the predominant type of leukocytes is neutrophil and also known as the first line of defense which enters the mammary gland from the blood [9,10]. The function of neutrophils is to engulf the microorganisms and kill them (phagocytosis). The release of milk SCC during infection as discussed above has been presented in Figure-2.

## Milk Differential Leukocyte Counts (DLCs)

Different patterns of leukocyte population of the healthy gland are a significant factor in mastitis. Thus, to analyze the heritability of a trait and its correlation with udder health, group of workers examined the leukocyte populations of uninfected mammary glands and found that the effect of the cow trait is significant for neutrophil, macrophage, and T-lymphocyte-bearing CD4+ [11]. Moreover, the patterns of leukocytes populations in milk together with the variance among cows can be used for the analysis of the heritability of this trait which can be correlated with udder health for future studies.

Morphological characteristics and DLCs of healthy and mastitis milk of different species have been presented in Table-1 [12-19]. There is an increase in the percentage of lymphocytes, followed by macrophages and neutrophils in normal mammary quarters [20]. However, reports of macrophage having the maximum percentage in normal milk SCC are also there [21,22]. Resident neutrophil in milk with low SCC might modulate the initial steps of dynamic immune defense of the udder, and percentage change in neutrophils in conjunction with SCC might provide a more reliable method for assessing the quality of milk in buffaloes [23]. Highest neutrophil percentage (36.91%) in colostrum at day 1 sample and the lowest (24.90%) at the 5^th^-day colostrum have been reported [24].

**Table-1:** Morphological characteristics and percentage of different leukocytes in healthy and mastitis milk of different species

Parameters	Neutrophils	Macrophages	Lymphocytes	References
Milk leukocyte of cow at 100× (Olympus IX51 microscope)	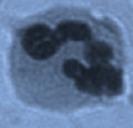	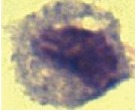	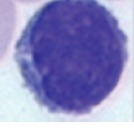	
Morphological characteristics	Diameter 12-15 μm, nucleus is multilobed with bridges	Diameter 20-30 μm, the largest cell type in milk	Diameter 9-16 μm, deeply stained round nucleus with little cytoplasm	Li *et al*.[12] Alhussien *et al*. [13]
Percentage of leukocytes in healthy and mastitis milk of different species				
Healthy cow	19	66*	15	Alhussien *et al*. [14]
Mastitis cow	75*	17	8	
Healthy buffalo	17	35	46*	Dang *et al*. [15,16]
Mastitis buffalo	67*	18	7	
Healthy ewe	31	57*	8	Morgante *et al*. [17]
Mastitis ewe	62*	31	5	
Healthy goat	79*	11	10	Boulaaba *et al*. [18]
Mastitis goat	?	?	?	
Healthy camel	9	66*	25	Hamed *et al*. [19]
Mastitis camel	60*	22	18	

* Predominant cell type

Lymphocytes were the predominant cell type in low and high SCC groups [25], but their proportion declined with the total BMSCC. Furthermore, the milk neutrophil may also be involved in generating a situation of oxidative stress in the mammary gland. The relationship between BMSCC, differential BMSCC, and lipolysis level was studied [26], and it was found that a positive relationship existed between total BMSCC, the neutrophil population, and lipolysis level in milk. Some researchers have found that lymphocytes represent the least percentage of SCC in both normal and mastitis milk [13]. Together these, leukocytes account for approximately 25% of SCs in “normal” milk and collectively function to detect and phagocytize pathogens and subsequently initiate an appropriate immune response. At the onset of infection, the resident macrophages and the mammary epithelial cells are the first cells to synthesize and release various pro-inflammatory cytokines such as tumor necrosis factor-alpha, interleukin-1, and interleukin-8 (IL-8). Chemoattractants like IL-8 induce chemotaxis of neutrophils from the surrounding blood vessels into the mammary gland. The lymphocytes coordinate the immune system response by the release of cytokines, and both macrophages and neutrophils play a role in the phagocytosis and destruction of bacteria. All these immune cells come from the blood into the udder as a surveillance mechanism to look for bacteria, which is a major factor contributing to the incidence of mastitis in dairy animals [27]. Differentiation of cells in milk is more beneficial and gives a definite description of the actual udder health status of cows. However, its practical application for dairy herd improvement testing programs needs further investigation [28].

## Factors Affecting Milk SCC

Many factors such as milk productivity, health of an animal, management, and environment affect the release of SCs in milk as discussed below.

### Effect of productivity on milk SCC

A relationship exists between SCC and milk yield, although the heritability may be low. High milk-producing cows are under stress of milk production, and their immunity becomes low leading to more SCC in their milk [29]. High milk SCC not only negatively affects milk yield but also milk composition and quality [30], and younger cows due to their low milk-producing ability have lower SCC [31].

### Effect of different stages of lactation on milk SCC

Lactation of a dairy cow can be divided into early, mid, and late lactation. Milk yield is highest during early lactation and then decreases subsequently with the progress of lactation. Effect of the stage of lactation was significant for all lactation age groups, (≤2 to ≥6) and SCCs were highest shortly after calving, declined rapidly to a nadir between days 25 and 45 and then rose slowly throughout the rest of lactation cycle [32]. There was a strong correlation between SCC and stage of lactation, SCC of healthy quarters increased from approximately 80×10^3^ cells/ml at 35 days postpartum to 160×10^3^ cells/ml at 285 days postpartum [33].

The mean SCC values in the milk of first lactation were higher during early lactation (1.10-1.27×10^5^ cells/ml), which decreased during mid-lactation (0.90-0.99×10^5^ cells/ml), and increased marginally during late lactation (0.99-1.07×10^5^ cells/ml) in buffaloes [34]. Overall, mean SCC values in cows were higher during the 1^st^ month of lactation which decreased during the 2^nd^ month and after that fluctuated up to day 300 of lactation [35]. The milk losses per unit increase of log-transformed SCC (LnSCC) in Holstein cows were estimated throughout the lactation cycle [36]. Milk loss was high during early lactation and declined in mid-lactation, and highest milk losses were observed during late lactation.

Milk SCC was recorded in Belgian dairy heifers during early lactation and estimated for their impact on test-day SCC. The geometric mean of SCC (5-14 day in milk) of the 14,766 available samples was 104,000 cells/ml and decreased from 178,000 cells/ml at the 5^th^ day to 74,000 cells/ml at the 14^th^ day in milk [37]. The stage of lactation had a clear effect on IMI; the least risk of IMI was in the 1^st^ month of lactation and maximum during the 10^th^ month with an approximate difference of 6.3 fold in the probability of IMI. The prevalence of IMI quickly increased reaching a maximum of 79% during the 3^rd^ month of lactation and then decreased slightly to 60% during the mid-lactation (6^th^ month) before increasing to about 75% during late lactation in buffaloes [38]. Mammary glands of high yielding cows have a better magnitude of innate immune response during mid-lactation stage as compared to early and late lactation regarding milk SCC and *in vitro* immune response of isolated milk leukocytes [39]. The integrated effects of seasons and stage of lactation on SCC in Sahiwal cows have been studied [27]. It was found that milk SCC was maximum in early lactation, decreased in mid-lactation, and rose again in the late lactation. Although both stages of lactation and season had significant (p≤0.001) effects on the SCC, their interactions did not affect SCC.

Factors affected by the incidence of elevated cow composite SCC (≥200,000 cells/ml) at first test milking after first calving dairy heifer were investigated in southwest Sweden [40]. It was found that 18.1% of the animals had elevated SCC at first test milking (21 days) after calving. The other factors associated with increased risk of elevated SCC were increasing amounts of concentrates moving to confined housing on the day of calving instead of earlier and use of restraint measures at milking.

### Effect of parity on milk SCC

Young primiparous cows produce less milk and have a lower milk SCC as compared to multiparous cows [36,41]. Milk SCC is affected by parity with first calvers having low SCC. The overall mean SCC was 3.95 (51.9×10^3^ cells/ml), the least squares means of SCC for bacteriological negative in first, second, and third parities, respectively, were 3.80 (44.7×10^3^ cells/ml), 3.93 (50.9×10^3^ cells/ml), and 3.97 (53.0×10^3^ cells/ml) in cows [42]. However, it was seen that the paired comparison of bacteriologically negative cows in the second lactation versus the first lactation was borderline significant and third lactation cows versus the first lactation was not significant [43]. Another study conducted observed that the natural log of SCC from uninfected quarters of first parity cows was highest during the first part of the lactation [44]. It was also seen that the mammary gland immunity of primiparous cows is always higher as compared to the multiparous cows throughout the lactation period [45]. Recently, the diurnal variation of milk SCC in Karan Fries cows during different parity (1, 2–4, and >4) was recorded and no difference was seen in the milk SCC up to fourth parity; however, it increased significantly (p<0.05) in cows having more than four parity. Although milk SCC was similar in the morning and evening samples of all groups, cows with more than four parities exhibited a significant diurnal variation in the DLC and were more prone to udder infection [46]. Furthermore, the reaction of the milk SCs to pathogens may increase with age which makes them more prone to new infections. There may even be longer infections and more tissue damage in older cows.

### Effect of body condition score (BCS) and body weight on milk SCC

BCS is a method used to evaluate fatness or thinness in cows according to a given defined scale. An increase in the BCS at calving was associated with reduced somatic cell score (SCS) in first- and second-parity cows and greater SCS in cows of third parity or greater [47]. They found that increased BCS and body weight loss during the early stage of lactation were associated with less SCS and a lower probability of a high test-day SCC. They further reported that the body weight was positively associated with SCS although the effect was greater in Jersey cows than in Holstein-Friesians. They also reported that several body weight variables were positively associated with a greater likelihood of clinical mastitis (CM).

### Effect of season on milk SCC

Environmental factors significantly influence milk SCC in a herd. This is because extreme temperatures not only impose stress on the animals but also influence the intake of feed. High humidity in some seasons like hot-humid and some micronutrient deficiencies due to the poor quality of fodder may also cause more growth of the infectious bacteria accompanied with low immunity. BMSCC is highest in spring and summer in those countries where calving patterns are non-seasonal and is possibly related to the influence of higher temperature and humidity on the risk of IMIs [48]. The highest BMSCC around the period of calving was observed in the winter, and the lowest BMSCC in these herds occurred shortly after the calving period [49]. Higher levels of SCC during hot-humid season in elite cows compared to non-elite cows indicate more stress level on the udder of these animals during this particular season [50]. In milk, casein (except γ-CN) was lower in the summer and higher during the winter season. IgG and serum albumin contents were higher in summer than in winter and spring seasons [51]. A mild effect of season was also observed by them for milk SCC, with greater values in summer than in the winter and spring seasons. Milk coagulation properties were worsened during the summer season. Values of milk SCC and neutrophil: macrophage (N: M) ratio were lowest during thermoneutral, intermediate in winter, and highest during the summer season. Milk SCC and N: M ratio exhibited a diurnal rhythm in both cows and buffaloes during summer season [46,52]. Therefore, there is an urgent need to record and tabulate day-to-day variation of milk SCC of all animals kept under natural conditions on a farm. Any deviation in these variations should be evaluated critically, and necessary management steps need to be followed to maintain optimum milk quality.

### Effect of milking on milk SCC

The removal of milk from the mammary glands of a cow either by the calf, by hand, or by machine is the three ways of milking in a dairy herd. With an increase in the milk-producing capacity of the animals, machine milking has been introduced in all big farms. Machine milking requires proper cleaning, smooth functioning, and maintenance according to the manufacturer specifications. The relationship between milk SCC and milking practices followed under organized farms, under peri-urban systems, and in single holdings under rural conditions was investigated [53]. A comparison was also made between samples collected from bucket-milked and hand-milked crossbred cows. Higher (p<0.01) values of SCC in hand-milked animals were found as compared to machine-milked cows. It was also observed that practices like post-milking teat dipping with antimicrobial solutions decrease the amount of SCC coming in milk in subsequent milkings. Modern automatic machines have inbuilt SCC online counters which can alert the dairy managers as soon milk passes through them. BMSCC was compared from 24 months before install of automatic milking system until 48 months post-installation [54]. It was seen that there are significantly higher levels of BMSCC during the 12-month post-installation. However, these decreased over time and even showed a significant lower BMSCC after 36 months post-installation. These findings indicate that automatic milking had a negative impact on milk quality during the early stage. However, once farmers become accustomed to managing the machine and the cows adapt the new technology which takes on average 188.4 days [55], the milk quality improves significantly.

### Effect of breed on milk SCC and DLC

**Figure-3:**
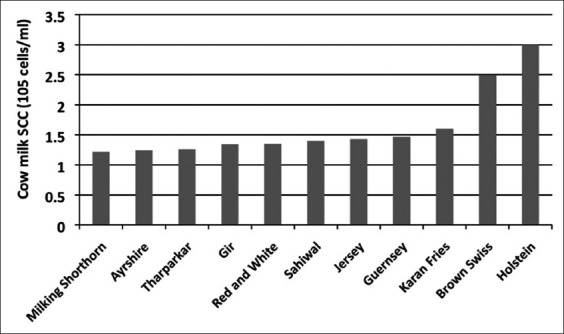
Milk somatic cell counts in different breeds of healthy cows.

SCC variation has been noted between various breeds of cows [14,22,27,56-58] as presented in Figure-3. The high-producing cattle breeds such as Brown Swiss and Holstein have a higher presence of SCC/ml in milk. Average SCC for 212 dairy herds was found to be around 241,000±83,000 cells per ml [42]. It has been found that indigenous cow breeds like Tharparkar have less milk SCC as compared to Karan Fries cows [14,22]. Breed type also influences the shape of udder, and teats with well-attached udders show less incidence of mastitis than pendulous-shaped udder [15,59]. Influence of the shape and size of teats have been seen, and it was found that milk SCC is always higher in shorter teats having a greater diameter of teat canal [60].

### Effect of physiological stage on milk SCC and DLC

The effect of various stressful physiological stages including subclinical mastitis (SCM), CM, pregnancy, and calving on both milk SCC and DLC in Karan Fries cows was investigated [14]. Maximum values of SCC were noticed in CM cows (7.50×10^5^ cells/ml), followed by SCM cows (4.60×10^5^), then in the colostrum (4.00×10^5^) collected on the day of calving, and finally, in pregnant cows (2.00×10^5^). Unlike SCC, the percentage milk neutrophils were maximum in CM, calving, and SCM cows, respectively. Interestingly, milk macrophage percentage was high in healthy animals (65.53%) and was significantly (p<0.05) low in case of both CM (16.59 %) and calving cows (19.32%). However, milk lymphocyte showed less fluctuation among the groups compared to neutrophils and macrophages.

### Effect of infection-causing pathogens on SCC

Two types of bacteria or pathogens (contagious and environmental) can be found in the mammary tissue of dairy animals. Contagious pathogens spread from cow to cow and environmental pathogens are present in the herd’s surroundings such as bedding materials, manure, and soil. *Staphylococcus aureus*, *Streptococcus agalactiae*, and S*treptococcus dysgalactiae* are classified as contagious pathogens, which can efficiently adapt to the environment of the mammary gland and spread from cow to cow during milking [2]. Pathogens such as *Streptococcus uberis, Enterococcus* spp., *Arcanobacterium pyogenes*, coagulase-negative *Staphylococci*, and coliforms are classified as environmental pathogens and considered as opportunistic pathogens of the mammary gland. These microbes are mainly transferred from the contaminated environment to the mammary gland of a cow at the time of milking. Interestingly, more research has being deviated recently to identify the relationship between the mastitis-causing pathogen and the molecular and cellular changes taking place in mammary tissue during any pathological infection. The intensity of udder inflammatory process is associated with the number of mastitis pathogens shedding from the infected mammary gland [61]. *S. aureus-*infected buffaloes had maximum milk SCC, followed by *Escherichia coli* and *S. agalactiae*. However, *S. agalactiae* pathogen was responsible for higher SCC compared with other pathogens in mastitis dairy cows [62]. The percentage of neutrophils in the mammary gland of buffalo was maximum during *S. agalactiae* infection, followed by *E. coli* and *S. aureus*. Furthermore, the level of mastitis did not affect blood counts, but it influenced the milk SCC of normal quarters [15]. The presence of *S*. *aureus* and *Arcanobacterium pyogenes* was considerably associated with high bulk tank SCC. Significant differences were found in the presence of *S. aureus, S. agalactiae, and S. dysgalactiae* in bulk tank milk sampled from the small household farms, dairy-farming communities, and large-scale dairy farms [63].

Dry period of 40 days is given to the cows during their lactation cycle. This period allows the mammary gland to be repaired and restored for the next lactation. This is the most critical period of lactation cycle, and precautions should be taken during this period as milk get accumulated in the udder and may leak from it. This leaked milk may act as a good culture media for bacteria which may enter the teat and then subsequently move into the upper parts of the mammary gland. Giving dry cow therapy during this period may reduce the infections and thus reduce milk SCC in the coming lactation [7].

### Effect of High Milk SCC on Milk Yield and Composition

The variation in milk quality and composition in relation to SCC has been presented in Table-2 [22] High milk SCC is caused by inflammatory conditions of the mammary gland which changes the milk composition of milk more like that of blood. This is caused by an increase in the permeability of blood mammary barrier which results in more ions, proteins, and inflammatory cells coming in milk. An increase in the milk SCC is associated with a decrease in milk yield. This decrease in the milk yield may be observed at least about 1 week before CM is diagnosed. This may be due to the fact that mastitis is subclinical before the onset of CM. Further, the effects of mastitis are severe if the infection occurs during early lactation and before the animal has reached its peak milk yield [64,65]. Furthermore, these cows produce less milk throughout the rest of lactation in comparison to the cows which were free from mastitis. Cows once infected do not return to their pre-mastitis milk yield level [66]. The decrease in milk yield with an increase in milk SCC is mainly due to physical damage to the milk-producing epithelial cells, and as a result, the synthetic and secretory capacity of the mammary gland will considerably reduce. Many inflammatory mediators change in stimulatory or inhibitory hormone concentrations, and reduced availability of milk precursors may play a major role in the reduction of milk production during mastitis [67]. Furthermore, more energy gets deviated toward the immune system rather than milk production, and the animal may eat less due to pain and decreased movements.

**Table-2:** Variations in the milk quality of cow in relation to SCC [22].

Milk constituents	Healthy	Subclinical mastitis	Clinical mastitis
SCC (10^5^cells/ml)	<2	3-5	>5
Fat (%)	4.32	4.31	4.08
Protein (%)	3.30	3.34	3.70
Casein	2.70	2.55	2.25
Whey protein	0.84	1.13	1.35
Serum albumin	0.17	0.24	0.37
Lactose (%)	4.84	4.71	4.41
SNF (%)	9.73	9.61	9.35
pH	6.61	6.63	6.80
EC (mS/cm)	5.90	6.01	7.21
Chloride	0.09	0.13	0.16
Sodium	0.05	0.09	0.11
Potassium	0.18	0.16	0.13

SCC=Somatic cell counts, SNF=Solids-not-fat

Milk SCC had a significant effect on the milk yields, milk protein, and milk lactose but not on the milk fat composition [30]. An increase in protein but a decrease in lactose percentage was observed in the milk of infected cows [22]. Lactose is responsible for 50% of the osmotic pressure of milk and a decrease in its level causes a dramatic reduction in milk yield, and more ions shift from blood to milk to maintain the osmotic balance. Milk from healthy cows has approximately 80% protein as casein and 20% as whey protein. Reports indicate that there is a change in the protein profile of mastitis cows and they have a rise in the level of whey proteins. However, they may show a decrease in α- and β-caseins. These changes may be due to the regulation of the lactation-related genes in response to infection and from the induced hydrolysis of milk protein observed when the milk SCC slightly increases in both SCM and CM [66]. Moreover, an increase in proteins is due to disruption to the integrity of the mammary epithelium by microbial toxins and the opening of the tight junctions. Damage to the mammary epithelium causes an influx of blood-borne proteins into the milk. There is an increase in the caseinolytic enzyme in milk, plasmin. Plasmin is derived from plasminogen which originated from the blood and probably leaks into the milk due to the disruption of the epithelium. Plasmin cleaves β-casein into smaller casein and polypeptide fragments which then diffuse into milk. This leads to poor curding, lowered cheese yield, and a bitter taste of dairy products.

The decrease in the fat concentration of mastitis cows may be due to reduced synthetic and secretory capacity of the mammary gland. An increase in free fatty acids is observed due to alteration in the milk fat globule membrane by the enzyme lipases produced by leukocytes or by plasmin through the hydrolysis of lipoproteins. However, some researchers have reported a decrease in the lipoprotein lipase activity in mastitic milk, whereas report of a rise in lactoferrin, protein content, and plasmin and a decrease in casein/protein ratio, calcium, and phosphorus in *S. aureus* CM is also there [68].

SCM reduced lactose, solids-not-fat (SNF), and total solid content, but no significant difference was observed in the protein and fat content between infected and non-infected quarters [69]. The decline in milk lactose has been attributed due to the damage to alveolar epithelial cells. Furthermore, there is a leakage of lactose out of the milk through the paracellular pathways that proliferate during mastitis. Potassium leaks through the paracellular pathway, and therefore, its concentration decreases. Conversely, sodium and chloride found in high quantity in blood leak into the milk and increase above normal concentration.

In general, total solids and SNF had a slight increase, whereas fat content had a significant increment. However, the more intense changes occurred in the proteins [70]. The crude protein had a small elevation in milk with SCC around 700,000 cells/ml and decreased with SCC above 1,000,000 cells/ml. Percentage of casein reduced and that of soluble proteins decreased which led to a considerable reduction of casein: soluble protein ratio. Higher milk SCC also caused a reduction in β-, α-, and κ-casein fractions. Unlike previous findings, high bulk tank milk SCC exhibited non-significant effects on the percentage of total proteins and lactose [71].

Milk SCC has a negative influence on milk powder quality [72] and other technological traits of milk [30,73]. Research conducted also indicated that plasminogen activator activity per cell was approximately 4 fold higher in the intermediate SCC group when compared to the low SCC group [74]. Many recent studies have shown that SC positively influences the technological properties and quality of cheese through the release of their endogenous enzymes [12,75]. However, further studies are required to see the relationship between the amount of milk SCC, their enzymes, and its subsequent effects on the milk traits.

### SCC and DLC in Fraction-Collected Milk

Total SCC in various fractions of milk collected during milking was studied to understand the cellular variation in various fractions of milk [76]. The first five fractions did not differ from primary milk but stripping and complimentary milk each contained more SCs than primary milk. Correlation between total SCCs in each fraction with the total SCC in primary milk ranged from 0.91 to 0.97. The percentage of milk granulocytes was 66.4, 68.5, 66.8, 69.2 % and mammary epithelial cells was 27.6, 24.9, 24.3, 21.1 % of the total SCs in the first 20-ml, ninth 20-ml stripping, and complementary milk, respectively. The variation in milk SCC and DLC in different strips (early, mid, and late) in buffaloes was investigated. Early and mid strips of milk had lower and similar levels of SCC, and their levels increased significantly in the late strip. The neutrophil percentage was maximum during the early strip, intermediate in the mid-strip, and minimum in the late strip. Although milk lymphocytes were higher in mid-strip compared with early and late strips, the percentage of milk macrophages was similar in all fractions of milk [52].

### SCC and DLC in Colostrum

Colostrum is the first milk produced during the 1^st^-week post-calving, is very rich in immunoglobulins, and provides immunity to the newborn calf. The variation in milk SC in primiparous cows during the first 75 days of lactation was evaluated [77]. SCC was highest in colostrum and lowest at 9-10 week in milk. For differential cell counts in milk, only the percentage of macrophages changed significantly during first 75 days (33% at 1 week, 25% at 6 weeks, and 34% at 11 weeks). Epithelial cells were found to be 11-20% of the total milk SCC. They reported that milk SCC variation between dairy cows within pairs sampled was around 3-24%. However, the variation was considerably greater for the DLC (46 % for lymphocytes and 34% for epithelial cells). Total milk SCC after first calving is not a stable characteristic of individual cows; however, it appears to depend mainly on differences in temporary factors. It was also found that proportions of the different SC types in milk may vary consistently by a cow during the early stage of the first lactation.

Variation of SCC in colostrum and milk of first and second lactation cows was also observed during 10 days after calving [78]. Mean foremilk SCCs were found to be higher than mean bucket milk counts in both the first and the second lactation. A negative correlation between SCC in both foremilk and bucket milk samples and milk yield was evident in both the groups of cows. There is higher SCC in colostrum of Murrah buffaloes which become normal by the 5^th^ day of milking [79]. In colostrum of cow, SCC is about 5.00×10^5^ which reduced to 2.93×10^5^ at the 7^th^-day post-calving and remained almost constant in the subsequent weeks of lactation [80].

### Scenario of Milk SCC in Buffaloes

Milk SCC has been studied extensively in buffaloes in Brazil [81,82] and India [83]. The ultrastructure of the buffalo mammary gland was studied for the first time [83], and it was observed that similar to cows, the change of milk SCC and immunity of the mammary gland in buffalo is disturbed during involution and postpartum [15]. SCC of buffalo milk increases significantly by day 21 of involution and the 1^st^ week prepartum; it is significantly higher around parturition and became normal at 14-days postpartum. Therefore, the chances of udder infection during the dry period are low. However, once the buffaloes are milked after calving, loosening of the teat sphincter occurs due to continuous synthesis and removal of milk.

SCC of buffalo is always found to be lesser than cows [24,84]. Going a step further, isolation of milk macrophages, neutrophils, lymphocytes, and epithelial cells was done from total milk SCs of buffaloes. It was found that estimating the immune activity of individual SCs helps in better understanding of the immune regulation in the mammary gland [85]. Buffaloes with high SCC also have higher alkaline phosphatase activity in their milk [86]. As in cows, variation in the day-to-day levels of milk SCC has also been observed in Murrah buffaloes [52].

### Scenario of Milk SCC in Other Animals

SCC of camel milk has not been studied extensively; as results, the accepted “normal” value of camel has not been established [87]. SCC in camel milk can be used to diagnose CM or SCM [88], and it was found that in dromedary milk, SCC decreases significantly with the stage of lactation [33]. However, some researchers reported that the seasonal variation in milk quality indicators mainly SCC and total viable count in bulk camel milk is the consequence of seasonal reproduction and lactation curve characteristics of dromedaries [89]. A higher prevalence of *S. aureus* and *Streptococcus* species in the milk of camel with higher SCC was observed, and it was recommended to implement teat dipping, and herd level mastitis treatment as strategies to lower SCC and milk losses [90].

Small ruminants such as sheep and goat give less milk, and chances of mammary infections are lesser in them as compared to cows. In this study, a total of 2159 samples of ewe milk were taken and divided into five groups based on SCC: Low <2×10^5^ cells/mL, middle=2-4×10^5^ cells/mL, higher=4-6×10^5^ cells/mL, high=6-10×10^5^ cells/mL, and mastitis >10×10^5^ cells/mL [91]. The percentage of ewes according to the SCC classification was as follows: 71.79%, 10.24%, 5.05%, 4.03%, and 8.89%, respectively. Moreover, 82.03% of samples had SCC below 4×10^5^ cells/mL and only 8.89% over 10×10^5^ cells/mL [91]. Sheep milk with SCC>1,000,000 decreased the quantity of cheese yield and increased the development of rancid flavors in the cheese samples [92]. Therefore, more research is needed to define the acceptable sheep milk SCC for optimal cheese processing in sheep milk. Cells are found to be higher in goat milk due to the presence of non-leukocytic cell-like fragments in its milk. In caprine milk, a level of 7.0×10^5^ cells/ml represented the threshold for fluctuations in leukocyte distribution, which is mainly related to the immune status of the udder [93]. The non-infectious factors such as parity and stage of lactation did not affect milk SCC for cows and sheep but had a major impact on the counts for goats and therefore needed to be considered when establishing legal limits for the goat milk [56]. However, in another study, the quality of goat milk was less affected by SCM and high milk SCC as compared to that of bovines [94].

### Methods of Estimating Milk SCC

#### Direct microscopic method

**Figure-4:**
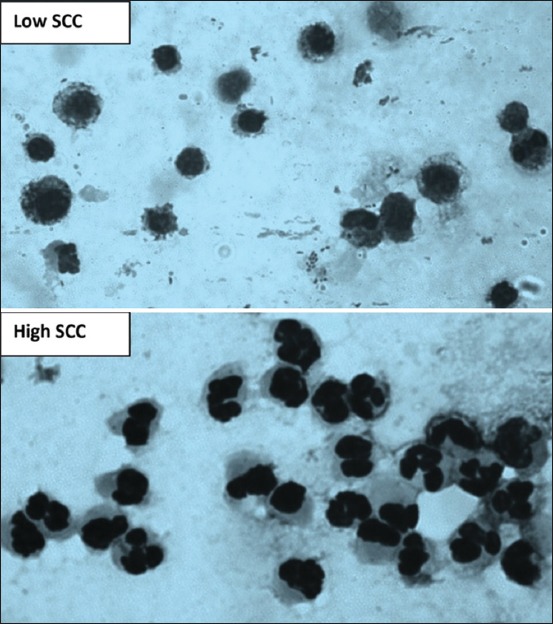
Microscopic examination of milk somatic cells smear showing that macrophages are the major cell in low somatic cell counts (SCCs) and neutrophils are the main cell in high SCC.

Direct microscopic somatic cell count (DMSCC) method is an easy to use and cheap method which can be used by trained technical person/veterinarian under the field conditions. In this method, fresh milk is collected, and about 5-10 μl of milk is spread on a 1 cm^2^ (1 cm×1 cm) area of a degreased microscopic slide and is dried in a horizontal position. The films are air-dried with 96% ethyl alcohol for 3 min, air dried again, defatted with xylene for 10 min and rinsed with 60% ethyl alcohol, air dried and stained with methylene blue dye for 15 min. The slides are then rinsed with water and air-dried again. DLCs are usually used to study the presence of various cell types such as lymphocytes, neutrophils, and macrophages in the milk samples. For viewing and differentiating various cells, the focus is fixed and constant adjustment of the microscope’s fine focus is done [7]. SCs in low and high milk SCC of cows estimated by the direct microscopic method are shown in Figure-4.

For estimating SCC from goat and sheep milk, there is a problem in differentiating between cells and cytoplasmic particles. In goats, the presence of numerous non-cellular particles in milk due to apocrine type of milk secretion, which differs from the merocrine type of cow milk secretion, further exacerbates this problem. Consequently, DNA-specific stains such as pyronin Y-methyl green are used [95]. In animals which have more fat or solids in their milk, slides are treated with poly-l-lysine and dried for a longer time to improve adhesion. The rules for identifying and counting SCs have been given by the FDA (https://foodsafety.foodscience.cornell.edu/sites/foodsafety. foodscience.cornell.edu/files/shared/documents/CU-DFScience-Notes-Milk-Somatic-Cell-Counting-06-10.pdf).

#### Use of electronic cell counters

A significant disadvantage of DMSCC analysis is the tendency to color artifacts and with the potential problem of cell aggregation, and limited sample volume gives rise to uncertainty in the number of cells. A portable lactoscan for estimating milk SCC has been developed by many companies. It uses a very sensitive fluorescent dye for capturing to make the cell analysis more accurate, reliable, and fast. To count the SC with an electronic counter, the milk sample is mixed with the dying reagent, containing fluorescent dye like Sofia Green. Only 12 µL from the dyed sample is pipetted on the measuring chamber of a disposable chip. The chip is loaded into the device, and for a period of few seconds to 2 min, depending on the measuring mode, the analysis is done. SCC system focuses automatically on the chip, and the dyed cells are captured by the sensitive CCD camera. The analysis algorithm of digital images determines the number and dimension of the fluorescent cells and counts their concentration. The results automatically display on the screen and also on the printer, with the possibility to save the results and generate a report. Recently, flow cytometry approach to simultaneously identify cell types of bovine milk using specific antibodies has been used which appear to be a reliable method over the sample microscopic method [96]. To make the counting of milk SCC uniform throughout the world, the International Dairy Federation and International Committee for Animal Recording have launched a new project, which intends to set up an international reference system for SCs in raw milk. Quick SCC app has been developed for I phones. It allows taking real-time images of actual milk samples directly in the app and saves time, and the reports can be sent instantly to the laboratory.

### How to Reduce Milk SCC at Farm Levels

**Figure-5:**
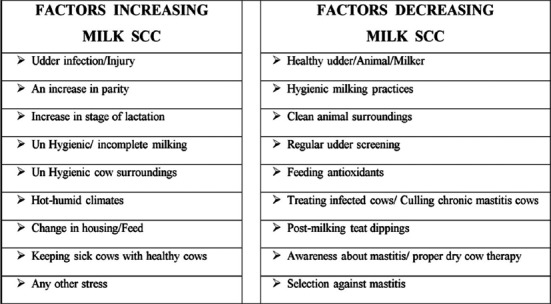
Diagrammatic representation of various factors and management practices that modulate the number of somatic cells in a dairy herd.

There are many factors and management practices that affect the release of milk SCC and can cause a decrease or increase in their levels as indicated in Figure-5. Researchers over the years have found associations between various management practices adopted on dairy farms and herd SC counts [97,98]. It has been reviewed by many authors that some of the precautions carried out during milking procedures: Wearing gloves during milking, using automatic takeoffs, using post-milking teat dipping, milking affected cows at last, inspection of the milking system at yearly intervals, and keeping the cows to stand following milking; all practices were consistently associated with lower herd SCC. Free stall system, providing sand bedding, cleaning the calving pen after each calving, monitoring of dry cow udders for mastitis, use of blanket dry cow therapy, supplementation of micronutrients, udder hair management, and frequent testing by California Mastitis Test all are associated with lower SCC. Regarding SCC of heifers, most of the consistent associations reported were related to interventions made during the peripartum period [99]. Recently, the association between mastitis control measures and BMSCC was investigated in Swedish dairy herds [100]. Two management practices, i.e., providing mineral feed and using teat disinfectant were found to be significantly associated with udder health.

### Use of Omics for improving milk yield and quality

Application of genomics, transcriptomics, proteomics, and metabolomics in biological research is known as Omics. It is the collective characterization and quantification of pools of biological molecules that get translated into the structure, function, and dynamics of an individual organism. Various studies have been conducted on the Omics of cow lactation to observe the loci associated with average SCs estimated throughout the lactation length with the test day SCS. Wijga *et al*. [101] found that SNPs on *Bos taurus* autosome 4 (BTA4) and BTA18 were significantly associated with average lactation SCC and BTA6 was found to be associated with standard deviation of test-day SCS. Sundekilde *et al*. [102] used nuclear magnetic resonance metabonomics to study the association between milk metabolites and SCCs. They found that metabolites such as hippurate and fumarate were associated with lower milk SCC, whereas lactate, butyrate, isoleucine, acetate, and β-hydroxybutyrate were associated with high values of SCC. RNA sequencing was performed to study the transcriptomic changes of milk SC isolated from mammary gland of sheep experiencing fish oil-induced milk fat syndrome [103]. They found that this syndrome is coupled with downregulation in mammary lipogenesis-associated genes and upregulation of genes involved in signal transduction and codification of transcription factors. Li *et al*. [104] highlighted the importance of Omics in lactation research. They reviewed that biological information related to changes observed in gene, mRNA, and protein expression along with the milk metabolites during different physiological conditions will help in understanding the regulation of various physiological and pathological processes. Koeck *et al*. [105] demonstrated that patterns of milk SCC could provide valuable information for genetic evaluation of mastitis resistance in cows. The genetic correlations of CM with SCC and udder depth are highly strong [106,107]. Furthermore, many traits derived from milk SCC are genetically correlated with CM [108]. These traits include mean SCS, standard deviation of SCS as well as excessive test-day SCS pattern. In future, this information can help in the establishment of different genetic and genomic evaluation models which may lead to a more efficient selection of mastitis resistance cows.

### Use of vaccination and melatonin to lower milk SCC

The vaccine MASTIVAC I reduced the number of animals that became infected with *S. aureus*, improved the overall health of the mammary gland, and increased the quality and quantity of milk production [109]. This treatment also helped to eliminate the infection from already *S. aureus*-infected cows and reduced their milk SCC. However, polyvalent vaccine did not have any beneficial effects on the number of SCC in dairy animals infected with S. aureus [110]. Melatonin has been found to improve milk quality and enhance immunity of dairy animals. It was seen that cows treated with melatonin exhibited lower SCC and serum cortisol which might be due to the improvement of the immune status of these cows [111].

### Use of antioxidant supplementation to lower milk SCC and improve milk quality

Specific nutrients fed to the cow during its production cycle can influence the immune defenses of the cow. Apart from providing energy and protein, various macro- and micro-minerals along with vitamins should regularly be fed to the dairy cows. Antioxidants (both vitamins and minerals) protect the body from damage by the free radicals and have been used both in humans and animals to prevent or delay cell damage. The supplementation of dairy cows having high SCC with antioxidant vitamins such as Vitamin A, C, and E and β-carotene as well as antioxidant minerals such as selenium, zinc, and copper is very effective in reducing their SCC, normalizing their milk composition, and ensuring an early recovery from mastitis [80,85,112,113]. Supplementation of inorganic selenium reduced IMI in *S. aureus*-infected dairy cows as compared to non-supplemented animals [114]. Even the supplementation of a low diet with of Se (0.2 parts per million) reduces SCC in lactating dairy cows [115]. The Vitamin A plus β-carotene treatment reduced the levels of SCC, improved the udder health, and enhanced immunity of dairy animals. Supplementation of Vitamin E, Cu, and Zn around the transition period can efficiently reduce SCC in cows [116]. Vitamin E supplementation affects milk quality in two aspects: Reducing the SCC and the activity of the major proteolytic enzyme, named plasmin in milk [117]. It is clear that current knowledge is not conclusive regarding the amount of Vitamin E needed to maintain milk quality in terms of SCC or oxidation [118]. Finally, despite the large number of studies describing the beneficial role of various antioxidants on the quality of milk and its composition, still many researchers reported that dietary antioxidants had no effect on milk protein, lactose, fat, total solids, and non-fat solids of cow’s milk [119] or goat’s milk [120].

## Future Use of Milk SCC

To predict early detection in the fluctuations of milk SCC and their timely interpretation for obtaining better milk quality, various mathematical models have been developed which are cost-effective and can efficiently estimate milk quality parameters [121]. The use of new computer algorithms and adoption of online detection systems, and various biosensors have made early detection of mastitis very exciting, and these detection tools will be available in the next few years. But still, there is a need to develop methods to produce milk SCC reference materials for calibration of electronic SCC and to determine the effect of refrigerated storage and freeze-thaw stability of the milk SCC reference material used [122]. A new somatic cell count index has been recently developed by a group of workers [123]. It can be calculated as the differences between the measured values of the natural logarithm of SCC (ln(SCC)) plus the values for the standard shape of the SCC curve for a specific period, divided by the total area enclosed by the standard curve and upper limit of ln(SCC)=10 for milk SCC.

Some innovative work has been carried out by isolating various leukocytes and epithelial cells and studying their activity from the milk cell pellet after centrifugation at low speed. Gene expression carried out by isolating milk SC from the mammary gland helps in assessing the overall mammary gland immunity and may be used as a tool for genetic selection of high milk-producing animals with stronger immunity [124]. Successful isolation and culture of neutrophils, lymphocytes, and macrophages from colostrum and milk from buffaloes have been developed [85,125]. Milk-derived cells have been used for producing handmade cloned embryos of quality similar to that of skin-derived embryos although with a lower blastocyst rate [126]. Furthermore, when the RNA quality is conserved, mammary epithelial cells isolated from milk are a valuable, non-invasive source of mammary mRNA to study various factors that impact milk yield and composition, namely, once daily milking, feeding level, endocrine status, photoperiodic modulation, and stage of lactation [127]. In future, isolation and studies on cells coming in milk will open new avenues for treating mastitis and for studying the milk-producing ability of a cow.

## Conclusions

Milk SCs are an indicator of both resistance and susceptibility of dairy cows to mammary infections. This review highlights the requirement and importance of estimating milk SCC for improving the overall milk production of a dairy herd. We have seen that high milk SCC is undesirable from the standpoint of quality but maybe too much low SCC will make the cows more prone to mammary infections which need to be investigated by dairy researchers. Work carried out in our laboratory over the past 15 years indicates that irrespective of the breed, about 1 lakh SCs per milliliter of milk are always desirable. Milk SCC can be counted either microscopically or by electronic counters. Estimation of milk SCC microscopically can be successfully implemented under field conditions as it is simple, costs less, and has great potential to diagnose SCM when used in combination with the California mastitis test.

For the consumers, lower milk SCC means extended product shelf life and improved flavor. For farmers, low milk SCC means fewer treatments, lower costs and higher milk yield per cow. Developed countries use milk SCC for assessing udder health and milk quality on a regular basis, and once cows with high milk SCC are identified, further management steps are taken to lower milk SCC and reduce milk losses. Therefore, to improve the confidence of our consumers and sell our milk and milk products in the global marketplace, there is a necessity to follow all the control measures and set a legal limit for milk of our indigenous cows and buffaloes. There is also a need to incorporate the functional parameters of milk cells which may be a more effective tool to develop high milk yielding dairy cattle having more disease resistance. Further, the use of milk SCC as a management tool on a routine basis will help to maximize immunity and improve quality and quantity of milk as well as cow comfort and welfare.

## Author’s Contributions

Both authors have contributed equally to this review.
